# Current status and factors associated with social frailty in older adults with type 2 diabetes: a cross-sectional study

**DOI:** 10.3389/fpubh.2026.1891728

**Published:** 2026-07-23

**Authors:** Beibei Liang, Wenwen Liang, Ding Xu, Cuixia Lin

**Affiliations:** 1 School of Nursing, Shandong University of Traditional Chinese Medicine, Jinan, China; 2 Geriatrics Department, Qilu Hospital of Shandong University, Jinan, China; 3 College of Health, Shandong University of Traditional Chinese Medicine, Jinan, China

**Keywords:** associated factors, cross-sectional study, older adults, social frailty, type 2 diabetes

## Abstract

**Objective:**

This study aims to investigate the current situation of social frailty among older adults with type 2 diabetes, and to analyze the associated factors, in order to provide a scientific basis for the clinical formulation of precise nursing intervention measures.

**Methods:**

From February to May 2026, a convenience sample of 334 older adults with type 2 diabetes admitted to the Endocrinology Department of a Grade A Level III hospital in Shandong Province was selected as the study subjects. Data were collected using a demographic questionnaire, the HALFT, GDS-5, AIS, and APGAR scales. SPSS 26.0 was used to perform descriptive statistics, univariate analysis, Spearman’s correlation analysis, and binary logistic regression.

**Results:**

The prevalence of social frailty among older adults with type 2 diabetes was 26.3%. Univariate analysis revealed that age, educational level, marital status, monthly household income, residence, living alone, employment status, medical payment method, fall in the past year, diabetic complications, diabetic comorbidities, number of medications, physical frailty, exercise frequency, depression, sleep, and level of family support were significantly associated with social frailty (*p* < 0.05). Correlation analysis indicated that social frailty was positively correlated with depression and sleep, and negatively correlated with the level of family support (*p* < 0.01). Logistic regression analysis indicated that age 70–79 years, age ≥80 years, living alone, unemployment, physical frailty, and depression were statistically significantly associated with social frailty (*p* < 0.05).

**Conclusion:**

Social frailty among older adults with type 2 diabetes is at a moderately high level. Advanced age, living alone, unemployment, depression, and physical frailty are factors associated to social frailty. Healthcare providers should conduct early screening and implement personalized interventions for high-risk individuals to improve patients’ social functioning, support glycemic control, and promote healthy aging.

## 1 Introduction

As the population ages at an accelerating pace, the prevalence of type 2 diabetes among older adults has been rising year by year, becoming a major global public health challenge (1). According to statistics from the International Diabetes Federation, the number of adults aged 20–79 years with diabetes worldwide reached 589 million in 2024 and is projected to rise to 853 million by 2050 (2). The diabetes epidemic in China is particularly severe. Studies show that the number of people with diabetes aged 20 years and older in China has reached 233 million, with the age-standardized prevalence rising from 7.53% in 2005 to 13.67%, and projected to reach 29.10% by 2025 (3). Among these, the prevalence of diabetes among older adults is particularly striking. The population aged 60 years and older has exceeded 310 million, with approximately 78.9 million older adults living with diabetes, about 95% of whom have type 2 diabetes (4). As physical function declines and social roles shift, older adults with type 2 diabetes experience corresponding changes in their activities of daily living and social participation, making them prone to insufficient social engagement (5, 6). Therefore, older adults with type 2 diabetes must not only focus on physiological blood glucose control but also pay close attention to the complex interplay between this control and the maintenance of social functioning.

In this context, social frailty, as an important component of frailty, has gradually attracted increasing attention from scholars both in China and internationally and has become an emerging research focus in the field of aging. Social frailty refers to the state in which an individual is at increased risk of social vulnerability due to a sustained lack of social resources, social behaviors, social activities, and self-management abilities necessary to meet basic social needs (7). Previous research on social frailty has primarily focused on community-dwelling older adults or general older adult populations with chronic diseases. However, older adults with type 2 diabetes are not simply a subgroup of the general older adult population. This group faces multiple stressors related to blood glucose control, long-term medication use, dietary restrictions, risk of complications, and hospitalizations (8, 9). These factors not only affect their physical function and psychological wellbeing but may also contribute to social frailty by limiting outdoor activities, reducing social engagement, increasing dependency on care, and undermining confidence in self-management (10, 11). Therefore, analyzing the factors associated with social frailty in older adults with type 2 diabetes can help extend social frailty research from the general older adult population to specific clinical groups with complex chronic disease management needs.

At present, the current research pays relatively limited attention to the social frailty among older adults with type 2 diabetes, and there is a lack of relevant evidence for hospitalized patients. Hospitalized older adults with type 2 diabetes usually have more complex conditions and a heavier burden of complications, and their social participation and family support may differ from those of community-dwelling older adults. Therefore, by investigating the current status of social frailty among older adults with type 2 diabetes and analyzing its associated factors, this study aims to provide a scientific basis for targeted nursing interventions. It holds significant practical importance for reducing the risk of adverse outcomes among patients, alleviating the social and healthcare burden, and promoting healthy aging.

## 2 Methods

### 2.1 Study design and participants

This study used convenience sampling to select older adults with type 2 diabetes who were hospitalized in the endocrinology department of a tertiary hospital in Shandong Province from February to May 2026 as research subjects. Inclusion criteria: (1) Diagnosed as type 2 diabetes according to the 2026 version of “Clinical Guidelines for the Prevention and Treatment of Type 2 Diabetes in the Older adults” in China (4); (2) Age ≥ 60 years old; (3) Clear consciousness and clear language expression; (4) Understanding the purpose and significance of the study and voluntarily participating in this research. Exclusion criteria: (1) Malignant tumors, failure of important organs (heart, liver, lungs, kidneys) function; (2) Patients with acute complications such as diabetic ketoacidosis; (3) Incomplete medical records. Exclusion criteria: (1) Logical contradictions in the questionnaire responses, such as consistent options or options that do not conform to common sense; (2) Poor participation in the survey during the investigation, affecting the validity of the results, or those who withdrew during the investigation; (3) Patients with sudden changes in condition during the investigation. The researchers rigorously screened participants according to the inclusion and exclusion criteria and obtained informed consent from all participants before administering the questionnaire.

According to the descriptive research sample size estimation method proposed by Kendall (12), the sample size is 5–10 times the number of independent variables. In this study, 20 independent variables were included. The required sample size for the study should be 100–200 cases. Given a 20% invalid questionnaire rate, the sample size should be between 125 and 250 cases. Eventually, the study determined that 334 would be the actual sample size, and there were no missing values for the main research variables.

### 2.2 Research tools

#### 2.2.1 Social demographic characteristics questionnaire

This questionnaire consists of 16 items and is divided into three sections: sociodemographic data, disease-related data, and lifestyle habits. Sociodemographic data: age, gender, educational level, marital status, monthly household income, residence, living alone, employment status, and medical payment method. Disease-related data: duration of diabetes, fall in the past year, diabetic complications, diabetic comorbidities, polypharmacy, and physical frailty. Lifestyle and behavioral data: smoking status, alcohol consumption, and exercise frequency.

#### 2.2.2 Help, participation, loneliness, financial, talk scale

The HALFT scale was developed by Ma in 2018 based on longitudinal cohort study data on aging in Beijing (13). It is a simple self-reporting tool for screening social frailty in older adults. The Cronbach’s *α* coefficient of this scale is 0.60. The scale mainly consists of 5 items: ① Have you provided help to others in the past 1 year? ② Have you participated in group or leisure activities? ③ Have you felt lonely in the past 1 week? ④ Does your income meet the needs of daily life? ⑤ Do you have family members or friends to confide in? For the third question, a “no” answer is scored 0 points, and a “yes” answer is scored 1 point. For the remaining questions, a “no” answer is scored 1 point, and a “yes” answer is scored 0 points. The total score ranges from 5 points. A score of 0–2 indicates no social frailty, and a score of ≥3 indicates the presence of social frailty. This scale has been widely used among older adults in China, particularly in community-dwelling populations and hospitalized clinical patients (14, 15). In this study, the Cronbach’s *α* was 0.673.

#### 2.2.3 Geriatric depression scale-5

The GDS-5 scale is used to assess depressive symptoms in older adults and was simplified from the GDS-15 by Hoyl (16). This scale consists of 5 items. Each item is scored as 1 point if answered “yes” and 0 points if answered “no.” In this assessment scale, the first item is reverse-scored, and the total score ranges from 0 to 5. Higher scores indicate more severe depressive symptoms. If the score is ≥2, it indicates that the individual has depressive symptoms. The Cronbach’s *α* coefficient of this scale is 0.812, with a specificity of 81% and a sensitivity of 94%. In this study, the Cronbach’s α was 0.729.

#### 2.2.4 Athens insomnia scale

The AIS scale is an internationally recognized self-assessment tool for sleep quality, developed by scholars such as Soldatos (17). It is mainly used to evaluate the subjects’ insomnia status and sleep quality over the past month. This scale has good reliability and validity, with a Cronbach’s *α* coefficient of 0.904. The scale consists of 8 items, including sleep onset time, number of nighttime awakenings, total sleep time, sleep quality, and other measures. Each item is scored 0–3, with a total score of 0–24. A higher total score indicates poorer sleep quality. The specific scoring criteria are as follows: a total score of less than 4 points indicates no sleep disorder, 4 to 6 points indicate possible insomnia, and a score of more than 6 points indicates insomnia (18). In this study, the Cronbach’s *α* was 0.857.

#### 2.2.5 Family adaptation, partnership, growth, affection, and resolve

The APGAR scale was developed by Smikstein (19) to assess subjects’ satisfaction with family functioning and to objectively reflect the degree of care the family provides to the subjects. The Cronbach’s *α* coefficient of this scale is 0.833. This scale consists of 5 items: adaptability, cooperation, length, emotionality, and intimacy. Each item has a score range of 0 to 2 (almost never, sometimes like this, often like this), and the higher the score, the better the family care. A total score of 0 to 3 indicates severe dysfunction of the family function, 4 to 6 indicates moderate dysfunction, and 7 to 10 indicates good family function. In this study, the Cronbach’s *α* was 0.798.

### 2.3 Data collection

Before the investigation, all the researchers were uniformly trained to master the methods for completing the questionnaires and the evaluation standards for each scale, ensuring the consistency of the investigation. Subsequently, they contacted the relevant hospital management departments and obtained the consent and support of the head nurse of the endocrinology department. During the investigation, the researchers actively explained the purpose and significance of the research to the patients and could distribute the questionnaires only after the patients signed the informed consent form. They guided the patients in completing the questionnaires and patiently answered their questions. Patients could complete the questionnaires themselves. If they were unable to do so, the researchers used the inquiry method, recorded patients’ responses to the questionnaire items, and assisted them in completing the questionnaire. During the process of collecting the questionnaires, we will immediately check for any incomplete entries or those that do not comply with the filling requirements. We will also, on the premise of fully respecting the patient’s wishes, ask them if they are willing to supplement or modify the information. This is to ensure the completeness and accuracy of the data and to promptly eliminate invalid questionnaires. Patients have the right to refuse to answer any questions and can withdraw from the survey at any time. Refusing to supplement or withdrawing will not affect their medical treatment and care services. For information that patients clearly refuse to answer or cannot confirm, there is no mandatory requirement to complete it.

### 2.4 Statistical analysis

Data processing and analysis were conducted using SPSS 26.0 software. Descriptive statistical analysis was employed to describe the overall distribution of social frailty in older adults with type 2 diabetes: measurement data that followed a normal distribution were expressed as mean ± standard deviation, while measurement data that did not follow a normal distribution were represented by median and interquartile range, and count data were described by frequency and percentage. In the single-factor analysis, based on the data type and the results of the normality test, when the measurement data followed a normal distribution and the variances were homogeneous, independent sample *t*-tests or analysis of variance were used; when they did not, the Mann–Whitney U test or Kruskal-Wallis H test were adopted; count data were analyzed using the *χ*^2^ test to compare the differences in the detection rates of social frailty among different groups. Spearman correlation analysis was used to explore the correlations between depression, sleep, and family care with social frailty. The variables with statistical significance in the single-factor analysis were included in the multivariate binary Logistic regression model. All categorical variables were encoded using dummy variables, and a clear reference group was set up to identify their independent associated factors; *p* < 0.05 indicated statistical significance. Collinearity was assessed by variance inflation factor (VIF), VIF < 5 as no severe collinearity was present. The Hosmer-Lemeshow test was used to evaluate model fit, *p* > 0.05 indicated good fit, and the Nagelkerke *R*^2^ was also analyzed in combination.

### 2.5 Ethical statement

This study was approved by the Ethics Review Committee of the Second Affiliated Hospital of Shandong First Medical University (2025-12099). The study was conducted in accordance with the principles of the Declaration of Helsinki. Written informed consent was obtained from all participants after they had received a full explanation of the study, ensuring autonomous decision-making and voluntary participation.

## 3 Results

### 3.1 Basic characteristics of the participants

A total of 360 questionnaires were distributed, of which 334 were valid, resulting in a 93% recovery rate. The average age of older adults with type 2 diabetes was 68.32 ± 6.08 years; 49.1% were male (164) and 50.9% female (170). Most participants had junior high school (29.9%) or high school/technical secondary education (27.8%), were married (81.1%), had a monthly family income exceeding 5,000 yuan (50.0%), lived in urban areas (71.9%), were not living alone (83.8%), were retired (68.9%), and had city medical insurance (58.1%). Regarding disease-related characteristics, most participants had a diabetes duration of 5–10 years (32.6%), had not experienced a fall in the past year (81.7%), had 1–2 diabetic complications (47.9%), had 1–2 comorbidities (50.6%), were taking five or more medications (38.6%), and were physically frail (62.0%). Regarding lifestyle behaviors, most participants had never smoked (65.3%) or consumed alcohol (59.6%), and 47.9% reported no regular exercise. The GDS-5 score median was 1 (0, 2.25), AIS was 7 (4, 10), and APGAR was 7 (5, 9). Other details are in Table 1.

**Table 1:** General demographic data and univariate analyses (*n* = 334).

Variables	Total (*n* = 334)	Non-social frailty (*n* = 246)	Social frailty (*n* = 88)	*χ*^2^/*Z*	*p*
Age (years)				37.387	<0.001
60–69	202 (60.5)	169 (83.7)	33 (16.3)		
70–79	118 (35.3)	74 (62.7)	44 (37.3)		
≥80	14 (4.2)	3 (21.4)	11 (78.6)		
Gender				3.209	0.073
Male	164 (49.1)	128 (78.0)	36 (22.0)		
Female	170 (50.9)	118 (69.4)	52 (30.6)		
Education level				24.669	<0.001
Primary school or below	54 (16.2)	27 (50.0)	27 (50.0)		
Junior high school	100 (29.9)	71 (71.0)	29 (29.0)		
High school/technical secondary school	93 (27.8)	73 (78.5)	20 (21.5)		
Associate degree	47 (14.1)	39 (83.0)	8 (17.0)		
Bachelor’s degree or above	40 (12.0)	36 (90.0)	4 (10.0)		
Marital status				58.092	<0.001
Unmarried	2 (0.6)	1 (50.0)	1 (50.0)		
Married	271 (81.1)	223 (82.3)	48 (17.7)		
Divorced	13 (3.9)	7 (53.8)	6 (46.2)		
Widowed	48 (14.4)	15 (31.3)	33 (68.8)		
Monthly household income (RMB)				49.941	<0.001
<3,000	60 (18.0)	23 (38.3)	37 (61.7)		
3,000–5,000	107 (32.0)	81 (75.7)	26 (24.3)		
>5,000	167 (50.0)	142 (85.0)	25 (15.0)		
Residence				20.105	<0.001
Urban	240 (71.9)	193 (80.4)	47 (19.6)		
Rural	94 (28.1)	53 (56.4)	41 (43.6)		
Living alone				32.023	<0.001
Yes	54 (16.2)	23 (42.6)	31 (57.4)		
No	280 (83.8)	223 (79.6)	57 (20.4)		
Employment status				43.092	<0.001
Employed	31 (9.3)	29 (93.5)	2 (6.5)		
Resigned	6 (1.8)	4 (66.7)	2 (33.3)		
Retired	230 (68.9)	184 (80.0)	46 (20.0)		
Unemployed	67 (20.1)	29 (43.3)	38 (56.7)		
Medical payment method				20.754	<0.001
Self-paid	3 (0.9)	3 (100.0)	0 (0.0)		
New Rural Cooperative Medical Scheme	96 (28.7)	55 (57.3)	41 (42.7)		
Municipal health insurance	194 (58.1)	152 (78.4)	42 (21.6)		
Provincial health insurance	41 (12.3)	36 (87.8)	5 (12.2)		
Duration of diabetes (years)				5.431	0.143
<5	72 (21.6)	56 (77.8)	16 (22.2)		
5–10	109 (32.6)	82 (75.2)	27 (24.8)		
11–15	55 (16.5)	44 (80.0)	11 (20.0)		
>15	98 (29.3)	64 (65.3)	34 (34.7)		
Fall in the past year				23.032	<0.001
Yes	61 (18.3)	30 (49.2)	31 (50.8)		
No	273 (81.7)	216 (79.1)	57 (20.9)		
Number of diabetic complications				8.971	0.011
0	97 (29.0)	80 (82.5)	17 (17.5)		
1–2	160 (47.9)	118 (73.8)	42 (26.2)		
≥3	77 (23.1)	48 (62.3)	29 (37.7)		
Number of diabetic comorbidities				8.378	0.015
0	30 (9.0)	24 (80.0)	6 (20.0)		
1–2	169 (50.6)	134 (79.3)	35 (20.7)		
≥3	135 (40.4)	88 (65.2)	47 (34.8)		
Number of medications				10.122	0.006
1–2	89 (26.6)	75 (84.3)	14 (15.7)		
3–4	116 (34.7)	87 (75.0)	29 (25.0)		
≥5	129 (38.6)	84 (65.1)	45 (34.9)		
Physical frailty				15.650	<0.001
Yes	207 (62.0)	137 (66.2)	70 (33.8)		
No	127 (38.0)	109 (85.8)	18 (14.2)		
Smoking status				0.688	0.709
Never smoked	218 (65.3)	160 (73.4)	58 (26.6)		
Former smoker	42 (12.6)	33 (78.6)	9 (21.4)		
Current smoker	74 (22.2)	53 (71.6)	21 (28.4)		
Alcohol consumption				2.496	0.287
Never drank	199 (59.6)	148 (74.4)	51 (25.6)		
Former drinker	58 (17.4)	46 (79.3)	12 (20.7)		
Current drinker	77 (23.1)	52 (67.5)	25 (32.5)		
Exercise frequency (≥30 min per session)				12.464	0.002
0 times/week	160 (47.9)	105 (65.6)	55 (34.4)		
11–2 times/week	93 (27.8)	71 (76.3)	22 (23.7)		
≥3 times/week	81 (24.3)	70 (86.4)	11 (13.6)		
GDS-5 (scores)	1 (0, 2.25)	1 (0, 2)	3 (2, 4)	−10.470	<0.001
AIS (scores)	7 (4, 10)	6.0 (4, 9)	9.5 (6, 13)	−5.605	<0.001
APGAR (scores)	7 (5, 9)	7 (6, 9)	6 (4, 8)	−4.658	<0.001

### 3.2 The current situation of social frailty among older adults with type 2 diabetes and single-factor analysis

This study found that 88 older adults with type 2 diabetes experienced social frailty, with an incidence rate of 26.3%. The univariate analysis showed statistically significant differences for these factors: age, educational level, marital status, monthly household income, residence, living alone, employment status, medical payment method, falls in the past year, diabetic complications, diabetic comorbidities, number of medications, physical frailty, exercise frequency, depression, sleep, and level of family support (*p* < 0.05). In contrast, there were no significant differences in gender, duration of diabetes, smoking status, or alcohol consumption (*p* > 0.05). See Table 1 for details.

### 3.3 Correlation analysis of social frailty, depression, sleep and family care in older adults with type 2 diabetes

The Spearman analysis showed that HALFT was significantly positively correlated with GDS-5 (*r* = 0.631, *p* < 0.01) and AIS (*r* = 0.324, *p* < 0.01), and was significantly negatively correlated with APGAR (*r* = −0.317, *p* < 0.01). See Table 2 for details.

**Table 2:** Correlation analysis of social frailty with depression, sleep, and family caring degree.

Project	HALFT	GDS-5	AIS	APGAR
HALFT	1.000	0.631**	0.324**	−0.317**
GDS-5	0.631**	1.000	0.504**	−0.372**
AIS	0.324**	0.504**	1.000	−0.206**
APGAR	−0.317**	−0.372**	−0.206**	1.000

**p < 0.01.

### 3.4 Logistic regression analysis of the associated factors of social frailty in older adults with type 2 diabetes

Taking social frailty as the dependent variable and selecting the variables from the single-factor analysis that were statistically significant as independent variables, a Logistic regression analysis was conducted. The variable assignment method is shown in Table 3. The results showed that age 70–79 years, age ≥80 years, living alone, unemployment, physical frailty, and depression were significantly associated with social frailty. The collinearity test results show that the VIF values of all independent variables are all less than 3, indicating that there is no collinearity problem. The Hosmer-Lemeshow test showed that *χ*^2^ = 7.888, *p* = 0.444, indicating a good model fit. This model accurately predicted 89.2% of the study participants. Nagelkerke *R*^2^ = 0.702 indicated that the explanatory power of the model is at a relatively high level. Details are shown in Table 4.

**Table 3:** Assignment methods of independent variables.

Independent variable	Assignment method
Age (years)	60–69 = (0,0); 70–79 = (1,0); ≥80 = (0,1)
Education level	Primary school or below = (0,0,0,0); Junior high school = (1,0,0,0); High school/technical secondary school = (0,1,0,0); Associate degree = (0,0,1,0); Bachelor’s degree or above = (0,0,0,1)
Marital status	Unmarried = (0,0,0); Married = (1,0,0); Divorced = (0,1,0); Widowed = (0,0,1)
Monthly household income (RMB)	<3,000 = (0,0); 3,000–5,000 = (1,0); >5,000 = (0,1)
Residence	Urban = 0; Rural = 1
Living alone	No = 0; Yes = 1
Employment status	Employed = (0,0,0); Resigned = (1,0,0); Retired = (0,1,0); Unemployed = (0,0,1)
Medical payment method	Self-paid = (0,0,0); New Rural Cooperative Medical Scheme = (1,0,0); Municipal health insurance = (0,1,0); Provincial health insurance = (0,0,1)
Fall in the past year	No = 0; Yes = 1
Number of diabetic complications	0 = (0,0); 1–2 = (1,0); ≥3 = (0,1)
Number of diabetic comorbidities	0 = (0,0); 1–2 = (1,0); ≥3 = (0,1)
Number of medications	1–2 = (0,0); 3–4 = (1,0); ≥5 = (0,1)
Physical frailty	No = 0; Yes = 1
Exercise frequency (≥30 min per session)	0 = (0,0); 1–2 = (1,0); ≥3 = (0,1)
GDS-5	Original value entry
AIS	Original value entry
APGAR	Original value entry

**Table 4:** Logistic regression of factors affecting social frailty.

Variables	B	SE	Wald *χ*^2^	p	OR	95% CI
Age 70–79 years	1.517	0.563	7.252	0.007	4.559	1.511–13.755
Age ≥80 years	3.691	1.161	10.115	0.001	40.095	4.122–389.975
Living alone	1.740	0.605	8.270	0.004	5.695	1.740–18.639
Unemployed	3.247	1.266	6.577	0.010	25.708	2.150–307.417
Physical frailty	−1.381	0.622	4.932	0.026	0.251	0.074–0.850
GDS-5	1.588	0.264	36.176	0.000	4.895	2.918–8.214

To further assess the robustness of the results regarding the association between physical frailty and social frailty, this study conducted a sensitivity analysis. After verification, the coding of the physical frailty variable was correct, and no reverse coding or data entry errors were found. The diagnosis of multicollinearity shows that the VIF of this variable is 1.000, indicating no significant collinearity. In the unadjusted model, physical frailty was positively associated with social frailty (OR = 3.094, 95% CI: 1.740–5.503, *p* < 0.001). After adjustment for age, living alone, employment status, and depressive symptoms, the direction of this association became negative but was not statistically significant (OR = 0.486, 95% CI: 0.197–1.201, *p* = 0.118). In the fully adjusted model, physical frailty was negatively associated with social frailty (OR = 0.251, 95% CI: 0.074–0.850, *p* = 0.026). The above results indicate that the degree of model adjustment has a significant impact on the estimation of the association between physical frailty and social frailty. Details are shown in Table 5.

**Table 5:** Sensitivity analysis of the association between physical frailty and social frailty.

Model	Adjusted variables	OR	95% CI	P
Model 1	Unadjusted	3.094	1.740–5.503	<0.001
Model 2	Adjusted for age, living alone, employment status, and depression	0.486	0.197–1.201	0.118
Model 3	Model 3	0.251	0.074–0.850	0.026

## 4 Discussion

This study aims to analyze the current situation of social frailty among older adults with type 2 diabetes and the related factors. The results indicate a 26.3% incidence of social frailty, higher than the 14.1 to 25.3% reported in previous community studies (20–22), possibly because the sample comprised hospitalized rather than community-dwelling older adults. About 62% of patients experienced physical frailty, and nearly half had two or more diabetes complications. Acute stress from hospitalization, concerns about prognosis, and separation from familiar environments aggravate social resource loss and reduce social participation. Additionally, long-term dietary restrictions, medication compliance pressure, and limited activity due to complications together pose more obstacles to social interaction for these patients. Therefore, clinical staff should conduct routine screening for social frailty upon admission, provide social support and psychological counseling during hospitalization, and implement ongoing community-based health management after discharge to reduce social withdrawal and resource loss resulting from hospital isolation and disease burden.

This study showed that advanced age was significantly associated with social frailty. Compared with patients aged 60–69 years, patients aged 70–79 years and those aged 80 years and above were more likely to experience social frailty, this conclusion is supported by previous research results (23). A study by Cataltepe among older adults in Türkiye found that, with increasing age, older adults may experience reductions in social roles, the death of peers and relatives, declining mobility, and shrinking social networks, thereby becoming more vulnerable to insufficient social participation and reduced social support (21). However, in this study, there were only 14 patients aged ≥80 years. Although this group had a high OR and a wide 95% CI, this may indicate unstable parameter estimation due to sparse data. Therefore, the results for the ≥80 years group should be interpreted with caution. This study emphasizes the overall association between advanced age and social frailty, rather than the exact magnitude of the OR. Therefore, clinical medical staff should focus on managing hospitalized older adults with diabetes, providing active psychological care, facilitating reminiscence-based communication, and encouraging patients in the same room to communicate with each other, while assessing cognitive status and conducting simple cognitive interventions to delay social network atrophy and social functional decline associated with aging.

The solitary living status of older adults with type 2 diabetes is associated with social frailty. The risk of social frailty for those living alone is 5.70 times that of those not living alone. The impact of living alone on the health of older adults has received considerable attention. Studies have shown that living with family members can protect the health of older adults. Older adults who live alone report poorer health conditions and lower subjective wellbeing, but social participation can significantly reduce their vulnerability in terms of physical and mental health (24). In traditional Chinese family culture, the companionship and care provided by children are the primary source of social support for older adults. Older adults with diabetes who live alone lack timely emotional communication and health supervision in their daily lives (25). They may make mistakes in diabetes self-management behaviors, such as blood sugar monitoring, maintaining medication compliance, and implementing dietary control (26). This situation further leads to poor blood sugar control and triggers social withdrawal behaviors. A meta-analysis shows that the risk of type 2 diabetes in socially isolated older adults increases by 20%, and the risk of poor blood sugar control may also increase (27). This finding suggests that there may be a bidirectional reinforcing relationship between social isolation and diabetes, and the health risk of living alone may be further amplified in the diabetes population. Therefore, clinical medical staff should actively identify older adults with diabetes who live alone during rounds and care, and develop plans that include medication supervision reminders, remote blood glucose monitoring, discharge health education, and follow-up instructions to address deficiencies in family care.

The state of being unemployed is significantly associated with social frailty. Research shows that the risk of social frailty for the unemployed is 25.71 times that of the employed, while the risk for those who have left their jobs shows no significant difference from that of the employed. This suggests that the adverse impact of unemployment on health may not be solely due to the interruption of economic sources, but rather to long-term detachment from social life. Cho’s investigation also found that the unemployed state is significantly correlated with the risk of social frailty (20). Older adults with low socioeconomic status, due to insufficient resources to participate in paid social activities, face more pronounced restrictions on their social participation. Work is not only a source of economic income but also the main carrier of social identity recognition and daily social networks. Unemployed older adults may lack social connections and a sense of value recognition after leaving their social roles, and long-term social isolation may lead to deterioration in social functioning (28). In this study, the unemployed group was mainly composed of rural, low-income individuals. Among them, 42.7% used the new rural cooperative medical care payment method. The medical security level was low, and transportation conditions were limited, which restricted their ability to participate in social activities. Economic pressure and insufficient social resources led to a double deprivation. Therefore, clinical medical staff should pay special attention to older adults with type 2 diabetes who are unemployed. By assessing patients’ economic burden and social resources, free diabetes self-management group activities were held during hospitalization, and hospital volunteers were relied on to help patients establish low-cost social connections.

This study found that depression is positively correlated with social frailty, this finding is consistent with previous studies (29). The research shows that older adults with type 2 diabetes have a 60% higher risk of developing depressive symptoms compared to non-diabetic patients (30). Older patients with more pronounced depressive symptoms may exhibit reduced interest, less active communication, and decreased participation in social activities (31). Meanwhile, loneliness, insufficient social support, and reduced social participation, as reflected by social frailty, may also be associated with worsening depressive symptoms (32). In the context of the shrinking family structure and the gradual transformation of the older adults care model in Chinese society, there may be a bidirectional relationship between the depression problems and the social frailty states exhibited by older adults with type 2 diabetes. Therefore, clinical medical staff should incorporate depression screening into the routine assessment for older adults with type 2 diabetes upon admission, and promptly provide bedside psychological counseling or refer those with positive screenings to the hospital’s psychiatry department for intervention to prevent the mutual aggravation of depression and social frailty. This study used a cross-sectional design and therefore could not determine the temporal sequence or causal direction between the two conditions. Future longitudinal studies are needed to further explore the dynamic relationship between depressive symptoms and social frailty, thereby providing more reliable evidence for precise clinical interventions.

This study found that physical frailty was negatively correlated with social frailty, which was inconsistent with previous research results. Previous studies generally suggested that physical frailty and social frailty often coexist, and the decline in physical function may restrict an individual’s ability to engage in outdoor activities, participate in society, and integrate into the community, thereby being associated with a higher level of social frailty (33). However, the sensitivity analysis of this study revealed that the direction and statistical significance of the association between the two variables changed with the adjustment of covariates, suggesting that this result might be influenced by the model setup and the control of confounding factors, and its stability is limited. It may be related to the following reasons: Firstly, this study did not use a standardized scale for assessing physical frailty. Instead, it relied on a single question for patients to self-assess. Some patients who reported no physical frailty might have underestimated the actual degree of frailty due to having an overly high confidence in their own health status. In addition, the subjects of this study were older adults with type 2 diabetes who were hospitalized in tertiary grade A hospitals. Some patients with physical frailty might receive more family companionship, life care and medical attention due to the heavy burden of the disease, thereby improving their social support-related evaluations to a certain extent. This phenomenon is reasonable in the context of traditional Chinese family care culture and can be covered by family care for older adults. Although they show physical frailty, the loss of social resources for these patients can also be partially compensated. Although the collinearity diagnosis did not indicate significant multicollinearity, given the limited sample size and event count, as well as the change in effect direction after model adjustment, the interpretation of the negative correlation in the fully adjusted model still requires caution. Therefore, clinical healthcare professionals should adopt standardized tools for assessing physical frailty instead of relying solely on self-assessment. They should conduct objective rechecks for patients who report no symptoms of frailty but have suspicious findings from clinical examinations, and evaluate whether their family care networks actually provide sufficient social support.

## 5 Limitations

This study has several limitations. First, the cross-sectional design precludes causal inference about the relationship between variables and social frailty. Future longitudinal cohort studies are needed to examine causal relationships. Second, the study sample was drawn from a single tertiary Grade A hospital in Shandong Province. Patients treated in tertiary Grade A hospitals may have more severe disease, more comorbidities, and more complex health conditions; therefore, the findings may not be fully generalizable to community-dwelling older adults with type 2 diabetes or to populations in other regions. Multi-center and stratified sampling should be used in future research to include institutions at different levels and residential backgrounds. Third, social frailty was assessed using the HALFT scale, for which Cronbach’s *α* in the present study was 0.673, indicating acceptable but relatively low internal consistency. This may have affected the precision of social frailty measurement. Fourth, factors such as cognitive function, health literacy, diabetes self-management, digital literacy, social media use, and nutritional status may also be related to social frailty. However, due to the absence of relevant measurement tools during the design stage of this study, it is currently impossible to analyze the role of these factors. Future research should further integrate these variables to more comprehensively explain the formation mechanism of social frailty in older adults with type 2 diabetes. Fifth, due to the limited number of social frailty events and the inclusion of multiple variables, the model may carry a certain risk of overfitting; therefore, future validation through larger sample sizes and multicenter studies is recommended. Sixth, there were only 14 patients aged ≥80 years. The high OR and wide 95% CI observed in this age group suggest limited stability of the estimate. Future studies with larger sample sizes, particularly those including more patients aged ≥80 years, are needed to further validate this finding. Seventh, physical frailty is assessed with a single self-report item rather than a standardized, validated physical frailty scale, potentially reducing accuracy. Future studies should employ standardized, validated assessment tools to re-evaluate this relationship.

## 6 Conclusion

Social frailty is moderately high among older adults with type 2 diabetes. Advanced age, living alone, unemployment, depression, and physical frailty are associated factors for social frailty in older adults with type 2 diabetes. Clinical nurses should screen for mental health issues, assess social support, and create targeted interventions for high-risk older adult with diabetes. These steps help delay social frailty, improve social function, health, and quality of life, and support healthy aging and refined chronic disease management.

## Data Availability

The original contributions presented in the study are included in the article/supplementary material, further inquiries can be directed to the corresponding author.
